# Virulence Factors Associated with Pediatric Shigellosis in Brazilian Amazon

**DOI:** 10.1155/2014/539697

**Published:** 2014-04-29

**Authors:** Carolinie Batista Nobre da Cruz, Maria Carolina Scheffer de Souza, Paula Taquita Serra, Ivanildes Santos, Antonio Balieiro, Fabio Alessandro Pieri, Paulo Afonso Nogueira, Patrícia Puccinelli Orlandi

**Affiliations:** ^1^Instituto Leônidas e Maria Deane—Fiocruz Amazônia, Rua Terezina 476, Adrianópolis, 69.057-070 Manaus, AM, Brazil; ^2^Programa de Pós Graduação em Imunologia Básica e Aplicada (PPGBA-UFAM), Avenida General Rodrigo Octávio 6200, Coroado I, 69.077-000 Manaus, AM, Brazil; ^3^Departamento de Ciências Básicas da Saúde, Universidade Federal de Juiz de Fora, Câmpus Governador Valadares, Rua Israel Pinheiro 2000, Bairro Universitário, 35010177 Governador Valadares, MG, Brazil

## Abstract

Shigellosis is a global human health problem and the incidence is highest among children. In the present work, main *Shigella* virulence genes was examined by PCR and compared to symptoms of pediatric shigellosis. Thirty *Shigella* isolates were identified from an etiologic study at which 1,339 children ranging 0–10 years old were enrolled. *S. flexneri* was the most frequent species reaching 60.0% of isolates, 22.2% were *S. sonnei*, and 6.6% were both *S. dysenteriae* and *S. boydii*. All *Shigella* infected children had diarrhea, but not all were accompanied by others symptoms of bacillary dysentery. Among major virulence genes, the PCR typing revealed *ipaBCD* was present in all isolates, followed by *IpaH7.8*, *set-1A*, *set-1B, sen/ospD3, virF,* and *invE*. The pathogenic potential of the ShET-1B subunit was observed in relation to dehydration (*P* < 0.001) and ShET-2 related to the intestinal injury (*P* = 0.033) evidenced by the presence of bloody diarrhea. Our results show associations among symptoms of shigellosis and virulence genes of clinical isolates of *Shigella* spp.

## 1. Introduction

*Shigella* spp. is Gram-negative bacilli of the Enterobacteriaceae family that are perfectly adapted to colonize the host intestine subverting the host's defenses in their favor [1–4].

The genus* Shigella* encompasses four subgroups historically treated as species:* Shigella flexneri*,* Shigella boydii, Shigella sonnei*, and* Shigella dysenteriae* [5]. These species are the etiological agents of bacillary dysentery or shigellosis, manifested by fever, small volume of bloody, mucoid stools; abdominal cramps; and mucoid, bloody diarrhea [1, 6]. Other clinical manifestations range between nausea, vomiting, and dehydration. Depending on the virulence potential of the strain and the nutritional status of the individual, shigellosis can progress to severe disease when accompanied by rectal tenesmus, with neurological symptoms such as headache and lethargy [1].

*Shigella* virulence is based on the presence of a large virulence* inv* plasmid, carrying an operon that encodes the type III-secretion-system (T3SS) responsible for bacterial entry [7, 8]. The* ial* gene is found on* inv* plasmid and invasion-related processes [9]. The T3SS is composed of several proteins, including a needle shape oligomer anchored in the protein complex which connects the inner and outer bacterial membranes. The tip of the needle is oligomer composed for invasion plasmid antigens,* ipaB*,* ipaC,* and* ipaD* [6–9]. The* ipaH* gene is present as multiple copies, five on large plasmid and seven on chromosome. One of five copies, the* ipaH7.8*, plays a role in modulating the inflammatory response elicited by infection and shares a conserved C-terminal novel E3 ligase (C-term-E3-ligase) and variable N-terminal leucine-rich repeat (LRR) domains [10].

Others genes are important bacterial pathogenicity factors in the intestinal tract, such as the enterotoxins that have significant enterotoxic activity* in vitro* when tested in rabbit ileal loops and Ussing chambers [1].* Shigella* strains produce distinct enterotoxins:* Shigella* enterotoxin 1 (ShET-1) chromosome encoded by* set1A* which is present in all* S. flexneri 2a*.* Shigella* enterotoxin 2 (ShET-2) encoded by gene* sen/ospD3 *located on a large plasmid associated with virulence of* Shigella* and found in many, but not all,* Shigella* of different serotypes and also in enteroinvasive* Escherichia coli* (EIEC) [9, 11]. And two distinct Shiga toxins (Stx-1 and Stx-2) are encoded by chromosomal genes and expressed by* S. dysenteriae* and similar to the Shiga-like toxins of enterohemorrhagic* E. coli* [1].

The mechanisms of main pathogenic factors of* Shigella *are well stablished; however, studies focusing association between pathogenicity factors and shigellosis symptoms in human are scarce [12, 13]. In this work, the major virulence genes of* Shigella* species derived from pediatric bacillary dysentery were examined for PCR and the goal of this study was to investigate the relationship with symptoms of shigellosis.

## 2. Material and Methods

*Patients and Samples.* During a period from August 2007 to December 2008, stool specimens were collected from 1339 children ranging 0–10 years old who sought treatment at three hospitals, in Manaus, in the center of Brazilian Amazon, and transferred to a clinical microbiology laboratory. An axillary temperature higher than 37.8°C was considered fever when determined at the time of clinical assessment or as reported by the child's guardian. Dehydration was diagnosed by the attending medical professional. The presence or absence of vomiting was reported by the individual responsible for the clinical evaluation. The child's guardian was first informed about the research and asked to participate by filling out a consent form and a case report form (Ethics Committee of the Federal University of Amazonas 266/206). The inclusion criteria were as follows: the age of the patients was in the range of 0–10 years old, the patients had diarrhea that lasted 7 days, and blood was evident by stool examination with a fecal occult blood (FOB) test using the Feca-Cult Kit (Inlab diagnostica). The present study was designed to isolate* Shigella* strains from clinical samples of patients with bloody diarrhea by culture methods and characterize them by appropriate biochemical and serological tests.

*Bacterial Culture, Isolation, and Antibiogram.* Lactose nonfermenting colonies were selected on MacConkey lactose agar (MC),* Salmonella-Shigella* (SS), and xylose lysine deoxycholate (XLD) agar, and Shigella species were identified by biochemical panel that consisted of EPM and MiLi-citrate. A total of 36 isolates of* Shigella *spp. were identified. The* Shigella flexneri* M90T was used as reference strains for comparison purposes. The antibiogram technique was performed as described by [14]. The following antibiotics were tested: amikacin (AMK), amoxicillin + clavulanic acid (AMC), ampicillin (AMP), ciprofloxacin (CIP), chloramphenicol (CLO), ceftriaxone (CRO), gentamicin (GEN), kanamycin (K), nalidixic acid (NAL), and tetracycline (TET).

*Serological Tests.* The* Shigella* strains were subcultured on MacConkey agar plates, and serological tests were performed by the slide agglutination method. The serotypes of all* Shigella* isolates were determined with commercially variable polyclonal antisera (Promicro-Brazil) against all* Shigella* serotypes, including* S. sonnei* 1 and 2, polyvalent* S. flexneri*,* S. dysenteriae* 2, and* S. boydii* 11.

*PCR Assays.* Each sample was submitted to PCR amplification with ten pairs of different primers (Table 1). For the detection of virulence genes, DNA was extracted from the samples using the phenol-chloroform method. Ten pairs of primers corresponding to the genus* Shigella* and two primers (*uidA* and* invE*) corresponding to invasion genes that are also found in* Escherichia coli* were used. The primers sequences used were obtained from Invitrogen, Brazil. Descriptions and the sequences of the PCR primers used in this study are given in Table 1. The primers for* ipaH7.8* annealed a specific region that overlapped two contiguous genes, LRR and C-term-E3-ligase genes. The primers for* ipaBCD* amplified a product from loci* Ipa* located upstream to* ipaB* gene. Amplification was performed in a thermocycler (Eppendorf, Germany) by the methods described by Aranda et al. [13] and Faruque et al. [15]. The expected sizes of the amplicons were ascertained by electrophoresis in 1.5% agarose gel with an appropriate molecular size marker (Promega, Brazil).

The reactions were performed under the following conditions: 40 ng of DNA, 5X buffer, 0.25 mM dNTPs, 2.5 mM MgCl_2_, 5 *μ*M of each primer, 2.5 U of high-fidelity Taq DNA polymerase (Invitrogen), and sterile deionized water in a total volume of 12.5 *μ*L. PCR was performed in a thermocycler (Eppendorf) and consisted of the following steps: 94°C for 3 minutes, followed by 30 cycles of 94°C for 30 seconds, varying annealing temperatures for each gene (Table 1) for 45 seconds, and 72°C for 1 minute and 30 seconds. The final extension step was performed at 72°C for 10 minutes, followed by cooling to 4°C. The fragments obtained were analyzed by horizontal electrophoresis on a 1% agarose gel at 100 V in TBE buffer. The gel was stained in a solution of ethidium bromide and visualized on a transilluminator.

*16S rRNA Gene Sequencing.* To confirm* Shigella* species identification, a region from 16S rRNA gene located between 530° to 1492° nucleotides was amplified using the primers forward 5′-TGA CTG ACT GAG TGC CAG CMG CCG CGG-3′ and reverse 5′-TGA CTG ACT GAG AGC TCT ACC TTG TTA CGM YTT-3′ [16, 17]. The reaction (50 mM MgSO_4_, 0.5 *μ*L of 10 mM dNTPs, 5 pmol of each primer, 1.25 U Platinum Taq DNA polymerase High Fidelity, 10x buffer) consisted of three cycles (1x 94°C for 2 min; 35x 94°C for 30 s; 58°C for 30 s; and 1x 68°C for 1 min). After edition, the taxonomic affiliation was performed with “Ribosomal Database Project II” database. A minimum of 75% similarity was considered for the encountered species.

## 3. Results

### 3.1. Diarrhea Symptoms Related to* Shigella* Infections

In the present study, thirty* Shigella* species were isolated from an etiologic study at which 1,339 children presenting with diarrhea over the period from August 2007 to July 2008.* Shigella* species were the fifth most common cause of diarrhea (2.2%), that were led by enteropathogenic* Escherichia coli* in 837 cases (62.1%), followed by 207 children with* Rotavirus* (15.4%) and 192 with* Salmonella* species (14.3%), and 34 cases* of Yersinia* species (2.5%). Protozoa infection was observed in 46 cases:* Entamoeba histolytica *was found in 16 cases, 14 for* Giardia lamblia*, 13 for* Entamoeba coli*, and 3 for* Balantidium coli*. Twenty-four children had diarrhea associated with worms, 9 for* Enterobius vermiculares*, 9 for* Ascaris lumbricoides*, 4 for* Ancylostoma* species, and 2 for* Trichiura trichuris*. And still, the diarrhea etiology of one hundred ninety-nine children was unknown.

Monoinfections among major groups of enteropathogens were found, bacteria (*N* = 867), rotavirus (*N* = 39), and intestinal parasites (*N* = 8). Several coinfections were also found; thirteen children were infected by enteropathogenic bacteria, rotavirus, and intestinal parasites. Enteropathogenic bacteria coinfected with rotavirus in one hundred sixty-eight cases or with intestinal parasites in forty-five children were found.

**Figure 1:**
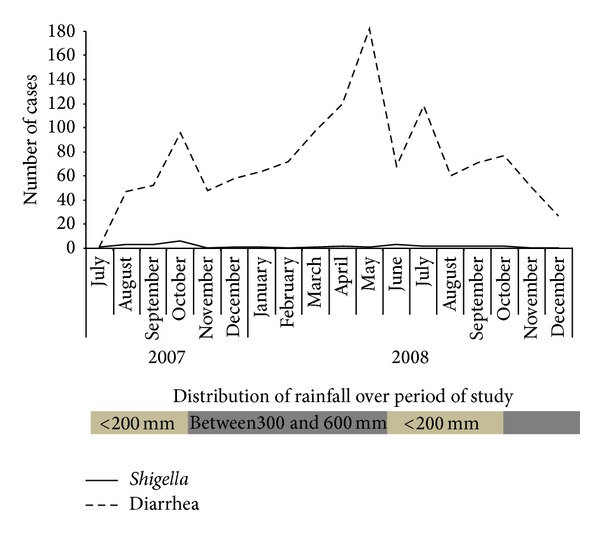
Temporal variation in diarrhea prevalence caused by* Shigella* and others enteropathogens. From August 2007 to December 2008, 1346 children in the range of 0–10 years old were admitted to hospital with diarrhea and they sought treatment at three hospitals in Manaus, in central of Brazilian Amazon. Stool specimens were collected at which* Shigella* as much as other enteropathogens were identified by classical methods. Distribution of rainfall over period of study is classified in two levels. Dark gray rectangles were the highest rate of rainfall (between 300 and 600 mm). Light gray indicates the rainfall that was below 200 mm [21].

Although rainfall in the region is seasonal [21], the temporal variation of cases of* Shigella* diarrhea did not fluctuate during the two rainfall stations, unlike the cases of diarrhea by other enteropathogens, which increased over the rainy season (Figure 1).

The study was carried out with children aged 0–10 years and as expected children over 2 years of age were moreaffected by* Shigella* (*P* = 0.002). The median of age of children affected by* Shigella* was 24 months (ranging from 14.2 to 47.2) differing from the group affected by other enteropathogens (14 months, ranging from 8 and 25). With respect to other epidemiologic factors, no difference was observed in both groups regarding the number and duration of diarrhea as well as the quality of the water consumed by population.

To characterize the main symptoms related to* Shigella* infections, initially the main diarrhea symptoms were compared among most prevalent etiologies (Table 2). The frequency of febrile children and dehydration signs were high and independent of etiology as expected. Similarly, the frequencies of children who have reported vomiting in clinical assessment were also high, except bacteria and rotavirus coinfected children whose frequency was slight higher (*P* = 0.006). In contrast, low frequencies of blood in stool and fecal occult blood were found among children independent of etiology, with even lower frequencies among coinfected children by rotavirus and bacteria or rotavirus monoinfection children (*P* = 0.009).

Regarding four enterobacteria, independently the analyses were performed with same symptoms. The frequency of febrile children and dehydration signs were high and independent of bacteria species or others etiologic agents. Also in relation to blood in stool, low frequencies and none difference were found. Differences were found regarding vomiting and fecal occult blood. Among* Shigella* infected children, the frequency of those who have reported vomiting in clinical assessment was lower in relation to others bacteria (*P* = 0.036) including coinfection groups (Table 2).

The main difference concerned fecal occult blood, while with all etiologic agents the presence of traces of blood in stool had been less frequent, and the number of* Shigella* infected children was higher than expected (*P* < 0.001). Thus, only with one accurate method traces of blood in stool might associate with bacillary dysentery (Table 2).

### 3.2. Virulence Genes Related to Pediatric Shigellosis

The conventional and 16S ribosomal gene confirmed 18 isolates of* S. flexneri* (8* S. sonnei*, 2* S. dysenteriae, *and 2* S. boydii* isolates). The antimicrobial resistance was 80.0% (24/30) to tetracycline, 40.0% (12/30) to ampicillin, 30.0% (9/30) to chloramphenicol, 30.0% (9/30) to gentamicin, and 13.0% (4/30) to both antibiotics amikacin and clavulanic acid. Thus, the resistance to ciprofloxacin and ceftriaxone was lower, with only 3% (1/30) of isolates presenting resistance. All isolates were sensitive to kanamycin and nalidixic acid (Table 3).

The detection of some major* Shigella* virulence genes gave intense amplicons with a clean background in each reaction according to conditions and PCR products (Table 1). The* ipaBCD* gene was present in all isolates. Concerning others virulence genes, a vast genetic diversity was shown among isolates;* ipaH* and* set-1A* genes were predominant in 63.3% of the isolates (19/30), followed by* set-1B* and* ial* in 56.7% (17/30) of the isolates (Table 3). The* sen/ospD3* (ShET-2),* virF,* and* invE* genes were present at a frequency of 43.3%, that is, in 13 isolates. Still, the* evt* was detected in 3 isolates (10.0%), despite the low frequency of* S. dysenteriae*. The presence of* evt* gene and antimicrobial resistance of the isolates are shown together with the symptoms presented by children (Table 3). Some isolates carried* set-1A* but not* set-1B*, or vice versa.

The high frequencies of* ipaBCD* and* ipaH* genes could explain frequencies of fever, vomiting, and dehydration in infected children. Regardless of* virF, invE,* and* evt* genes due low frequencies, the analyses were performed with* ial *and (invasion-related processes) and* set1-A* and* set-1B*. No association was found with fever, vomiting, or blood in stool with genes (data not shown).

In contrast, presence of blood traces in stool was related to shigellosis, and less common to all etiologic agents, two associations concerning* Shigella *enterotoxins were found. The* Shigella* species carrying* sen/ospD3* gene for ShET-2 enterotoxin hemolysin were more frequent in children that had traces of blood in stools (*P* = 0.042). And a strong association was found with dehydration and* set1-B* gene for* Shigella* enterotoxin 1 (*P* < 0.001) known for causing watery phase of diarrhea (Table 4). Thus, the PCR typing permitted us to connect particular virulence genes with symptoms of pediatric shigellosis.

## 4. Discussion

From a study in which the etiology of childhood diarrhea was investigated in 1,339 children from periphery of Manaus between August 2007 and July 2009, an intense and heterogeneous amount of enteropathogens found, from monoinfections to coinfections, were found in children from Manaus presenting with diarrhea. The lack of sanitation is a well-known problem in this city because less than 7% of the population has basic sanitation. Shigellosis is a disease that is one of the characteristics of areas like this, where it is difficult to maintain proper hygiene [1, 5, 12, 14, 15, 22–27]; thus, unsurprisingly the indicators of overall mortality and hospital morbidity due to diarrhea in Brazilian children are still worring [26].

What is interesting about findings on diarrhea-related symptoms is that independently if diarrhea was caused by mono- or coinfections, frequencies of febrile children, dehydration signs, and vomiting reported in clinical assessment were higher in all enteropathogens groups, and on the other hand frequencies of blood in stool among children were lower (Table 2). Moreover, detection of traces of blood in stool was in particular among* Shigella*-infected children. It is established that infection with* Shigella* can lead to the syndrome of bloody or watery diarrhea; nonetheless, studies, when the information of bloody diarrhea is reported by patients the frequencies, are divergent [28, 29]. Therefore, in the present study, the presence of blood in stool by more accurate method could be evidenced as a particular* Shigellosis*.

Shigellosis is an acute intestinal infection, the symptoms of which can range from mild watery diarrhea to severe inflammatory bacillary dysentery [3]. The thirty isolates of* Shigella* species were confirmed by conventional and 16S rRNA sequencing methods. Our data were consistent with observations in other regions of Brazil, with a predominance of* S*.* flexneri*, followed by* S. sonnei *or* S. boydii*, and finally* S. dysenteriae *[12, 15, 22–27, 30, 31].

Here, some isolates showed resistance to ciprofloxacin and ceftriaxone, which are the antibiotics recommended by the WHO for shigellosis. In contrast, in others studies conducted in North and Northeast of Brazil, all* Shigella* were susceptible to ciprofloxacin and ceftriaxone [24–27]. The emergence of resistant* Shigella* strains might be explained by the indiscriminate use of antimicrobial drugs or treatment failure. Even so, these data contribute to the monitoring of regional strains to ensure the effective treatment of patients and monitoring of the emergence of new resistant strains [24].

Despite the fact that* Shigella *species are considered as the important cause of diarrheal disease, little is known about their genetic diversity worldwide. According to virulence genes examined, the* Shigella *isolates in this study had a vast genetic diversity. Among main* Shigella* virulence factors, the T3SS is essential for host cell invasion and intracellular survival [32–34]. The presence of* IpaB*,* IpaC*, and* IpaD* translocators could be detected using the upstream* ipaB* region as marker. Our data revealed all the isolates were positive for the* ipaBCD* gene, as expected, whereas* IpaB*,* IpaC*, and* IpaD* are key factors of virulent* Shigella* [3]. Unlike* ipaBCD*,* ipaH 7.8 *was not very frequent. Because* ipaH 7.8* is present on a large plasmid, this gene would be less stable to storage/subculturing than chromosomal genes encoded by* ipaH*. [35]. Similarly,* ipaH *was detected in almost all Shigella species from western Brazilian Amazon [25].

Contingency analysis revealed* Shigella* carrying* sen/ospD3* was associated to fecal occult blood (*P* = 0.042). ShET-2 is known as an enterotoxin hemolysin that elicits inflammatory response during* Shigella* invasion. Our findings show that in cases of* Shigella *infection, ShET-2 contribute to induce intestinal injury induced by inflammation which would lead to bloody diarrhea [3, 8, 9, 11, 36, 37].

Regarding ShET-1 enterotoxin, contingency analysis showed* Shigella *isoletes that carry* set-1B *gene were associated with dehydration symptoms in children (*P* < 0.001). The ShET-1B subunit is enterotoxin, and according to experimental models, it alters the transport of water and electrolytes into the small intestine [1, 9, 38, 39]. Our findings confirm ShET-1B subunit as a potentially aggravating factor for dehydration in shigellosis.

## 5. Conclusions

We conclude that this PCR typing was able to identify irrespectively virulence genes in wild* Shigella* species, and our results showed vast genetic diversity of* Shigella* isolates. In addition, our study contributes to knowledge on particular symptoms of shigellosis associated with virulence genes, whose information about their roles are based on experimental models.

## Figures and Tables

**Table 1:** The striking points employed for the detection of virulence markers of *Shigella*.

Gene	Amplicon size (bp)	Primer	Annealing temperature °C	Reference
*evt *	100	CAACACTGGATGATCTCAG	56	[15]
		CCCCCTCAACTGCTAATA		
*ial *	320	CTGGATGGTATGGTGAGG	60	[18]
		GGAGGCCAACAATTATTTCC		
*ipaBCD *	500	GCTATAGCAGTGACATG	59	[15]
		ACGAGTTCGAAGCACTC		
*ipaH *	933	CTCGGCACGTTTTAATAGTCTGG	59	[19]
		GTGGAGAGCTGAAGTTTCTCTGC		
*set1A *	309	TCACGCTACCATCAAAGA	57	[18]
		TATCCCCCTTTGGTGGTA		
*set1B *	147	GTGAACCTGCTGCCGATATC	57	[18]
		ATTAGTGGATAAAAATGACG		
*sen/ospD3 *	799	ATGTGCCTGCTATTATTTAT	52	[18]
		CATAATAATAAGCGGTCAGC		
*virF *	618	TCAGGCAATGAAACTTTGAC	60	[19]
		TGGGCTTGATATTCCGATAAGTC		
*uidA *	1487	ATGCCAGTCCAGCGTTTTTGC	54	[20]
		AAAGTGTGGGTCAATAATCAGGAAGTG		
*invE *	766	CGATAGATGGCGAGAAATTATATCCCG	56	[20]
		CGATCAAGAATCCCTAACAGAAGAATCAC		

**Table 2:** Comparison of diarrhea symptoms among etiologic agents.

Symptoms	Bacteria monoinfection	Parasite and bacterial Coinfection	RV and bacterial coinfection	RV monoinfection	Unknown etiology	*P*	No bacteria as etiologic agent	*E. coli *	*Salmonella *	*Shigella *	*Yersínia *	*P*
	*N* = 867	*N* = 45	*N* = 168	*N* = 39	*N* = 199		*N* = 246	*N* = 837	*N* = 192	*N* = 30	*N* = 34	
Fever												
Pos.	646 (74.5)	31 (68.9)	127 (75.6)	34 (87.2)	148 (74.4)	0.185	189 (76.8)	615 (73.5)	144 (75)	25 (83.3)	28 (82.4)	0.588
Neg.	218 (25.1)	14 (31.1)	40 (23.8)	4 (10.3)	51 (25.6)		56 (22.8)	220 (26.3)	46 (24)	5 (16.7)	6 (17.6)	
NI^#^	3 (0.3)	0 (0)	1 (0.6)	1 (2.6)	0 (0)		1 (0.4)	2 (0.2)	2 (1)	0 (0)	0 (0)	
Vomiting												
Pos.	633 (73)	31 (68.9)	143 (85.1)	33 (84.6)	148 (74.4)	0.006	186 (75.6)	640 (76.5)	136 (70.8)	16 (53.3)	26 (76.5)	0.036
Neg.	231 (26.6)	14 (31.1)	24 (14.3)	5 (12.8)	51 (25.6)		59 (24)	195 (23.3)	55 (28.6)	13 (43.3)	8 (23.5)	
NI	3 (0.3)	0 (0)	1 (0.6)	1 (2.6)	0 (0)		1 (0.4)	2 (0.2)	1 (0.5)	1 (3.3)	0 (0)	
Dehydration												
Pos.	590 (68.1)	26 (57.8)	104 (61.9)	28 (71.8)	137 (68.8)	0.07	167 (67.9)	559 (66.8)	130 (67.7)	20 (66.7)	21 (61.8)	0.832
Neg.	239 (27.6)	19 (42.2)	54 (32.1)	9 (23.1)	60 (30.2)		72 (29.3)	238 (28.4)	55 (28.6)	10 (33.3)	12 (35.3)	
NI	38 (4.4)	0 (0)	10 (6)	2 (5.1)	2 (1)		7 (2.8)	40 (4.8)	7 (3.6)	0 (0)	1 (2.9)	
Blood in stool												
Pos.	134 (15.5)	3 (6.7)	20 (11.9)	3 (7.7)	38 (19.1)	0.312	46 (18.7)	118 (14.1)	24 (12.5)	10 (33.3)	5 (14.7)	0.074
Neg.	718 (82.8)	41 (91.1)	145 (86.3)	36 (92.3)	159 (79.9)		198 (80.5)	704 (84.1)	165 (85.9)	19 (63.3)	29 (85.3)	
NI	15 (1.7)	1 (2.2)	3 (1.8)	0 (0)	2 (1)		2 (0.8)	15 (1.8)	3 (1.6)	1 (3.3)	0 (0)	
Fecal occult blood												
Pos.	227 (26.2)	13 (28.9)	25 (14.9)	5 (12.8)	53 (26.6)	0.009	61 (24.8)	208 (24.9)	34 (17.7)	18 (60)	8 (23.5)	<0.001
Neg.	640 (73.8)	32 (71.1)	143 (85.1)	34 (87.2)	146 (73.4)		185 (75.2)	629 (75.1)	158 (82.3)	12 (40)	26 (76.5)	

Frequencies were calculated by the Chi-square test.

^#^NI: not informed.

**Table 3:** Frequencies and distribution of virulence genes and antimicrobial resistance of *Shigella* spp. and symptoms presented by children.

Isolates	*Shigella* species by 16S RNA gene	*ipaBCD *	*ipaH *	*set-1A *	*set-1B *	*Sen/ospD3 *	*ial *	*virF *	*evt *	*invE *	Antimicrobial resistance	Vomiting	Dehydration	Blood in stool	Fecal Occult Blood
2	*flexneri *	+			+		+						+	+	
53	*dysenteriae *	+							+		tet				
80	*flexneri *	+	+	+		+	+				amp, amk, amc, clo, tet	+			+
85	*flexneri *	+	+	+	+	+	+			+	amp, clo, tet		+	+	+
97	*flexneri *	+	+	+		+	+			+	amp, clo, tet		+		
113	*flexneri *	+	+			+					clo, tet	+			+
183	*sonnei *	+	+	+			+				gen, tet	+			
190	*dysenteriae *	+							+	+		+			
192	*boydii *	+			+		+	+	+	+	tet				
199	*flexneri *	+	+	+		+	+	+			tet	+			+
201	*flexneri *	+	+	+	+	+	+	+			amp, cef, tet		+		
202	*flexneri *	+	+	+							amp, tet				+
279	*sonnei *	+		+	+			+			tet	+	+		
337	*flexneri *	+		+	+			+		+		+	+		
539	*sonnei *	+		+			+				tet		+	+	
562	*sonnei *	+	+		+	+	+	+		+	gen, tet	+	+		+
586	*sonnei *	+	+	+		+	+	+		+	amp, amk, amc, clo, tet		+		+
625	*flexneri *	+	+	+	+	+	+			+	amp, cip, clo, tet		+	+	+
837	*flexneri *	+		+	+		+				tet	+	+	+	+
873	*flexneri *	+		+	+			+			tet		+	+	+
883	*sonnei *	+	+		+					+	amp, tet	+	+		+
893	*flexneri *	+	+	+		+	+				gen, tet				+
956	*boydii *	+		+	+	+	+	+		+	tet	+	+		+
1039	*flexneri *	+	+		+						amp, clo, tet	+	+	+	+
1065	*flexneri *	+			+						amp, clo,	+	+		
1118	*flexneri *	+	+	+	+	+	+	+		+	tet	+	+	+	+
1124	*sonnei *	+	+					+			amp, tet	+	+	+	+
1163	*flexneri *	+	+											+	+
1234	*sonnei *	+	+	+	+	+	+	+		+	amp, clo, tet	+	+		+
1257	*flexneri *	+	+	+	+			+		+	gen		+		

	Frequencies	100.0	63.3	63.3	56.7	43.3	56.7	43.3	10.0	43.3		53.3	66.7	33.3	60.0

+: Positive.

Abbreviations of antibiotics tested: amk: amikacin, amc: amoxicillin/clavulanic acid, amp: ampicillin, cip: ciprofloxacin, clo: chloramphenicol, cro: ceftriaxone, gen: gentamicin, and tet: tetracycline.

**Table 4:** Assessing of major *Shigella* virulence genes associated with main symptoms of dysentery bacillary.

Virulence gene	Dehydration		Prevalence ratio	CI	*P*	Fecal occult blood		Prevalence ratio	CI	*P*
	Pos.	Neg.				Pos.	Neg.			
*ial *										
Pos.	12 (60)	5 (50)	1.15	(0.68–1.94)	0.705	11 (61.1)	6 (50)	1.2	(0.65–2.22)	0.821
Neg.	8 (40)	5 (50)				7 (38.9)	6 (50)			
*ipaH *										
Pos.	16 (80)	8 (80)	1	(0.53–1.88)	0.999	16 (88.9)	8 (66.7)	2	(0.62–6.42)	0.184
Neg.	4 (20)	2 (20)				2 (11.1)	4 (33.3)			
*set.1A *										
Pos.	14 (70)	5 (50)	1.35	(0.74–2.47)	0.425	12 (66.7)	7 (58.3)	1.16	(0.61–2.19)	0.712
Neg.	6 (30)	5 (50)				6 (33.3)	5 (41.7)			
*set.1B *										
Pos.	16 (80)	1 (10)	3.06	(1.34–6.97)	<0.001**	10 (55.6)	7 (58.3)	0.96	(0.53–1.72)	0.999
Neg.	4 (20)	9 (90)				8 (44.4)	5 (41.7)			
*sen/ospD3 *										
Pos.	9 (45)	4 (40)	1.07	(0.65–0.77)	0.999	11 (61.1)	2 (16.7)	2.05	(1.11–3.80)	0.042*
Neg.	11 (55)	6 (60)				7 (38.9)	10 (83.3)			

*P* value of Fisher's exact test. **significant difference.
